# Interplay between Leucine-Rich Repeat Kinase 2 (LRRK2) and p62/SQSTM-1 in Selective Autophagy

**DOI:** 10.1371/journal.pone.0163029

**Published:** 2016-09-15

**Authors:** Sangwook Park, Seulki Han, Insup Choi, Beomsue Kim, Seung Pyo Park, Eun-Hye Joe, Young Ho Suh

**Affiliations:** 1 Department of Biomedical Laboratory Science, College of Health, Kyungwoon University, Gumi, 39160, South Korea; 2 Department of Pharmacology and Chronic Inflammatory Disease Research Center, Ajou University School of Medicine, Suwon, 16499, South Korea; 3 Department of Biomedical Sciences, Seoul National University College of Medicine, Seoul, 03080, South Korea; 4 Neuroscience Research Institute, Seoul National University College of Medicine, Seoul, 03080, South Korea; Niigata Daigaku, JAPAN

## Abstract

The deposit of polyubiquitinated aggregates has been implicated in the pathophysiology of Parkinson’s disease (PD), and growing evidence indicates that selective autophagy plays a critical role in the clearance of ubiquitin-positive protein aggregates by autophagosomes. The selective autophagic receptor p62/SQSTM-1, which associates directly with both ubiquitin and LC3, transports ubiquitin conjugates to autophagosomes for degradation. Leucine-rich repeat kinase 2 (LRRK2), a PD-associated protein kinase, is tightly controlled by autophagy-lysosome degradation as well as by the ubiquitin-proteasome pathway. However, little is known about the degradation of ubiquitinated LRRK2 via selective autophagy. In the present study, we found that p62/SQSTM-1 physically interacts with LRRK2 as a selective autophagic receptor. The overexpression of p62 leads to the robust degradation of LRRK2 through the autophagy-lysosome pathway. In addition, LRRK2 indirectly regulates Ser351 and Ser403 phosphorylation of p62. Of particular interest, the interaction between phosphorylated p62 and Keap1 is reduced by LRRK2 overexpression. Therefore, we propose that the interplay between LRRK2 and p62 may contribute to the pathophysiological function and homeostasis of LRRK2 protein.

## Introduction

The ubiquitin-proteasome system (UPS) and autophagy-lysosome pathway (ALP) are the major intracellular protein degradation pathways in eukaryotic cells. They were originally thought to function independently, however, accumulating evidence suggests that there is a crosstalk between these pathways with shared components [1–5]. Recent studies have indicated that several adaptor proteins, such as p62/sequestosome-1 (p62/SQSTM-1, hereafter referred to as p62), neighbor of BRCA1 gene 1 (NBR1), nuclear dot protein 52 (NDP52), and optineurin (OPTN) serve as selective autophagy receptors that link polyubiquitinated cargoes to the autophagic machinery [6–12]. These receptors contain a microtubule-associated protein 1A/1B-light chain 3 (LC3)-interaction region (LIR) and a ubiquitin-associated (UBA) domain, which binds to ubiquitin and to the mammalian Atg8 homologue LC3/GABARAP/Gate16 family, respectively [13, 14]. Among those receptors, p62 is the first selective autophagy receptor known to be responsible for the autophagic clearance of ubiquitin aggregates [13, 15].

The p62 protein is a multi-functional autophagy adaptor that was initially identified as a ligand of the Src homology 2 (SH2) domain of p56^lck^ [16]. p62 is a receptor for ubiquitinated substrates that are sequestered into autophagosomes, and it regulates protein aggregate formation [1, 2, 17]. Indeed, p62 is the major component of the ubiquitin-containing inclusions in various neurodegenerative diseases such as Parkinson’s disease (PD) [18, 19]. Moreover, loss of p62 suppresses the appearance of polyubiquitinated aggregates in autophagy-deficient mice [20]. However, the precise molecular mechanisms and pathophysiological roles of p62 in PD remain unknown.

Leucine-rich repeat kinase 2 (LRRK2) is a large, multi-domain protein with both GTPase and kinase activity [21–23]. Several mutations in LRRK2 have been identified as the most common genetic causes of PD. G2019S, the most prevalent mutation, enhances LRRK2 kinase activity, which is associated with neuronal toxicity and neurodegeneration. LRRK2 is degraded via the UPS by interacting with the carboxyl terminus of HSP70-interacting protein (CHIP), which consequently protects against cytotoxicity induced by LRRK2 [24, 25]. In addition, alterations in autophagy are consistently observed in the overexpression as well as the knockdown of LRRK2 [18, 24]. Recently, LRRK2 was found to be degraded in lysosomes through chaperone-mediated autophagy (CMA), whereas the G2019S LRRK2 mutant is more likely removed by the UPS and macroautophagy [26]. Nevertheless, the mechanism of LRRK2 stability regulation by selective autophagic receptors remains to be elucidated.

In the present study, we examined the functional role of p62, a representative selective autophagic receptor, in regulating the stability of LRRK2. We initially identified that p62 regulates LRRK2 turnover via autophagy-lysosomal degradation in heterologous cells and neurons. Then, we demonstrated that LRRK2 indirectly regulates the phosphorylation state and Keap1 binding of p62. Taken together, our data show that p62-mediated selective autophagy is necessary for LRRK2 degradation, which may underlie the pathogenesis of PD.

## Materials and Methods

### Ethics statement

The use and care of animals used in this study followed the guidelines of the Seoul National University Institutional Animal Care and Use Committee. Timed-pregnant Sprague-Dawley rats were obtained from the Orient Bio (Seongnam, Korea) and individually housed in standard cages during a period of acclimation with free access to food and water. Rats were kept in a controlled room at a constant temperature (22 ± 2°C) and humidity (50 ± 10%) on a 12 h light/dark cycle before used for experiments. Rats were sacrificed by CO_2_ asphyxiation followed by decapitation. Embryos were obtained by Caesarian section and decapitated. The protocol used specifically for this study was approved by the Seoul National University Institutional Animal Care and Use Committee (Permit Number: SNU-141231-2).

### Cell culture and antibodies

Human embryonic kidney (HEK) 293T cells were maintained in complete Dulbecco’s modified Eagle’s medium containing 10% heat-inactivated fetal bovine serum (Sigma Aldrich), 1 mM MEM non-essential amino acids solution, and antibiotics (mixture of 100 U/mL penicillin G sodium and 100 μg/mL streptomycin sulfate). Cells were grown at 37°C in a humidified atmosphere containing 5% CO_2_. The following antibodies were purchased from commercial sources: c-myc (9E10; Sigma Aldrich M5546), FLAG (M2; Sigma Aldrich F1804, F7425), α-tubulin (DM1A; Sigma Aldrich T6199), p62 (Abnova PAB16850), p62 phospho-Ser 351 (kindly provided by Dr. Komatsu, Tokyo Metropolitan Institute of Medical Science, Japan), p62 phospho-Ser 403 (4F6; Millipore MABC186), LRRK2 [MJFF2(c41-2); Epitomics 3514–1], LC3 (Abcam ab48394), Keap1 (Proteintech 10503-2-AP), green fluorescent protein (GFP, Invitrogen A11122), and horseradish peroxidase (HRP)-conjugated secondary antibodies (Invitrogen G21040).

### Plasmid construction

The deletion mutants of GFP-p62 were generated by a site-directed fragment deletion protocol as previously described [27]. Each primer pair contains partially complementary sequences at the 3’ end with extended non-overlapping sequences at the 5’ end. The oligonucleotide sequences for generation of GFP-p62 deletion mutants were as follows: ΔTB(p62 Δ225–251), forward: 5’-cccttgccccacagctgagtcgggcatcgaggttg-3’; reverse: 5’-catcaatgtcaacctcgatgcccgactcagctgtg-3’; ΔSMIR(p62 Δ166–224), forward: 5’-cctgcacagggagcacagcaaggcttctgctccat-3’; reverse: 5’-gatcctctgatggagcagaagccttgctgtgctcc-3’; ΔSMIR/TB(p62 Δ166–251), forward: 5’-cctgcacagggagcacagcaagggcatcgaggttg-3’; reverse: 5’- catcaatgtcaacctcgatgcccttgctgtgctcc -3’. The PCR reaction started with 3 min at 98°C and followed by 19 cycles of 30 sec at 98°C, 30 sec at 55°C, and 2 min 30 sec at 72°C. Following the PCR reaction, 1μL DpnI (New England Biolabs) was added and the mixture was incubated for 2 h at 37°C. 2μL of the mixture was transformed in DH5α competent cells by heat-shock, and *E*. *coli* was spread on LB agar plate containing 50μg/ml ampicillin. Plasmid DNA was isolated from colonies, and sequenced.

### Primary neuron culture

Primary rat cortical neurons were prepared from E18 Sprague-Dawley rats. Briefly, the cerebral cortices from rat embryos were dissected and incubated in Hanks’ balanced salt solution (Invitrogen) with 10 mM HEPES, 0.05% trypsin, 0.11 mg/mL deoxyribonuclease I, and penicillin-streptomycin for 12 min at 37°C. Trituration was performed 10~15 times with a fire-polished pasteur pipette. The dissociated cells were plated on poly-d-lysine-coated dishes in serum-free Neurobasal media (Invitrogen) containing supplemental B-27 and l-glutamine at 37°C in a humidified 5% CO_2_ incubator. Fresh media were added every 3~4 days.

### Viral transduction

Knockdown small hairpin RNA (shRNA) sequences targeting murine p62 (GCATCTACATTAAAGAGAA) [28] or rat LRRK2 (GTGATGTTTTCCTGTTAAT) were cloned under the H1 promoter of the pSuper vector (OligoEngine), and H1-shRNA sequences were cloned between the HIV-flap and ubiquitin promoter of the FUGW vector. To overexpress p62, we replaced GFP with p62 complementary DNA (cDNA) under the ubiquitin promoter of the FUGW vector. For the production of lentiviral particles, early-aged HEK 293T cells were co-transfected with FUGW lentiviral vector, packaging vector Δ 8.9, and vesicular stomatitis virus envelope glycoprotein vector by using X-tremeGENE (Roche). Supernatants were collected 48~60 h after transfection, aliquoted, and kept at –80°C until use.

### Western blotting and immunoprecipitation

HEK 293T cells or primary cortical neurons were washed three times with ice-cold 1 X PBS and solubilized in TNE lysis buffer (50 mM Tris-HCl, pH 8.0, 150 mM NaCl, 2 mM EDTA, 1% Triton X-100, and 0.1% sodium dodecyl sulfate) containing protease inhibitor cocktails (Roche). The lysates were incubated on ice for 30 min and centrifuged at 20,000 × *g* for 15 min at 4°C to remove insoluble materials. The supernatants were mixed with 6 X SDS-polyacrylamide gel electrophoresis (SDS-PAGE) loading buffer (375 mM Tris-HCl, pH 6.8, 12% SDS, 60% glycerol, 600 mM dithiothreitol, 0.2% bromophenol blue, and 15% β-mercaptoethanol), boiled at 95°C for 5 min, resolved with SDS-PAGE, and transferred to PVDF membranes. The membranes were blocked with 5% skim milk in 1 X TBST for 1 h at room temperature and then probed with the indicated primary antibodies overnight at 4°C. HRP-conjugated secondary antibodies were incubated with the membranes and detected by using an enhanced chemiluminescent substrate (Thermo Scientific). For immunoprecipitation experiments, the lysates were pre-cleared with Sepharose 4B beads (Sigma Aldrich) for 1 h at 4°C. The pre-cleared supernatants were incubated with antibody at 4°C for 1 h at 4°C and then with protein A- or G-Sepharose beads (Amersham) for 4 h at 4°C. The immunoprecipitates were then washed four times with lysis buffer and subjected to western blotting.

### Statistical analysis

Data are present as the mean and the standard error of mean (S.E.M) based on three or more independent experiments. Group differences were analyzed by the paired Student’s *t*-test or one-way ANOVA followed by Tukey’s post-hoc test. *P* value < 0.05 was considered statistically significant.

## Results

### LRRK2 can be degraded both in proteasomes and lysosomes in primary neurons

**Fig 1:**
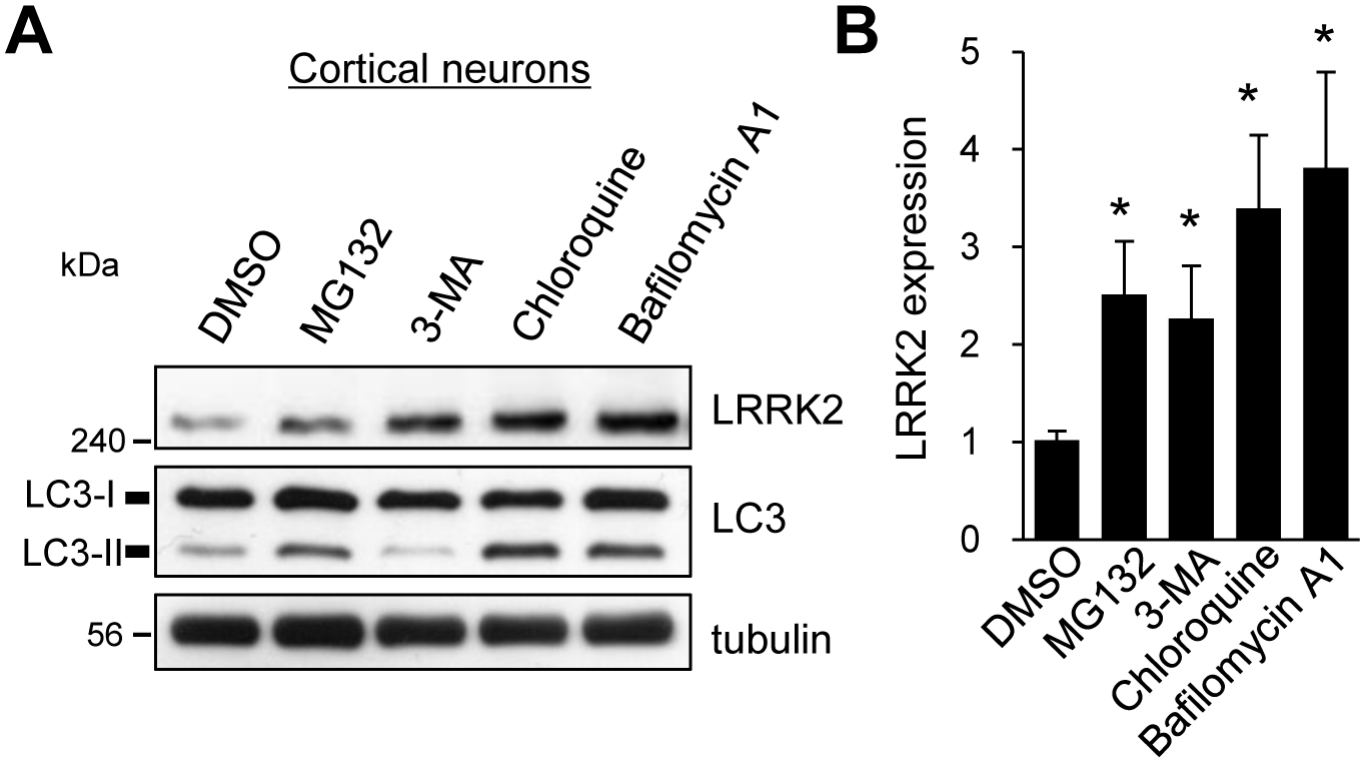
LRRK2 is degraded by both the UPS and the ALP. **A**. Primary rat cortical neurons at 14 days *in vitro* were incubated DMSO, 5 μM MG132, 10 mM 3-methyladenine (3-MA), 50 μM chloroquine, or 50 nM bafilomycin A1 for 2 h. Representative immunoblots for the indicated proteins from four to five independent experiments are shown. **B**. LRRK2 expression level relative to that in the DMSO-treated sample was quantified by using ImageJ software. Bar graph data represent the means of LRRK2 expression ± SEM (DMSO, 1.00 ± 0.08; MG132, 2.46 ± 0.52; 3-MA, 2.22 ± 0.51; chloroquine, 3.32 ± 0.70; bafilomycin A1, 3.73 ± 0.93; n = 4–5; *p < 0.05, paired *t*-test).

Impaired cellular proteolytic dysregulation caused by LRRK2 mutations and the consequent accumulation of aggregated proteins have been implicated in the pathogenesis of PD [26]. To investigate the degradation pathway of LRRK2 in neurons, we initially used several chemical inhibitors that specifically block the protein degradation pathway: MG132 (a proteasome inhibitor), 3-methyladenine (3-MA, a macroautophagy inhibitor), chloroquine (a lysosomal proteolysis inhibitor), bafilomycin A1 (an autophagosome-lysosome fusion inhibitor). At 14 days *in vitro*, the primary rat cortical neurons were incubated with the inhibitors. Compared with the DMSO control, treatment with MG132 for 2 h increased the total expression of LRRK2 in neurons by approximately 2.5-fold (Fig 1A and 1B). We also found that the lysosomal degradation of LRRK2 was blocked by treatment with chloroquine or bafilomycin A1, consistent with previous findings that LRRK2 is degraded both by the UPS and the ALP (Fig 1A and 1B) [24–26]. By contrast, LRRK2 protein levels were also significantly increased by treatment with 3-methyladenine, which blocks the macroautophagy pathway to lysosomal degradation. This finding indicates that neuronal LRRK2 can be degraded in lysosomes through macroautophagy, while Orenstein et al. showed LRRK2 can be degraded through the CMA [26].

### LRRK2 interacts physically with p62

**Fig 2:**
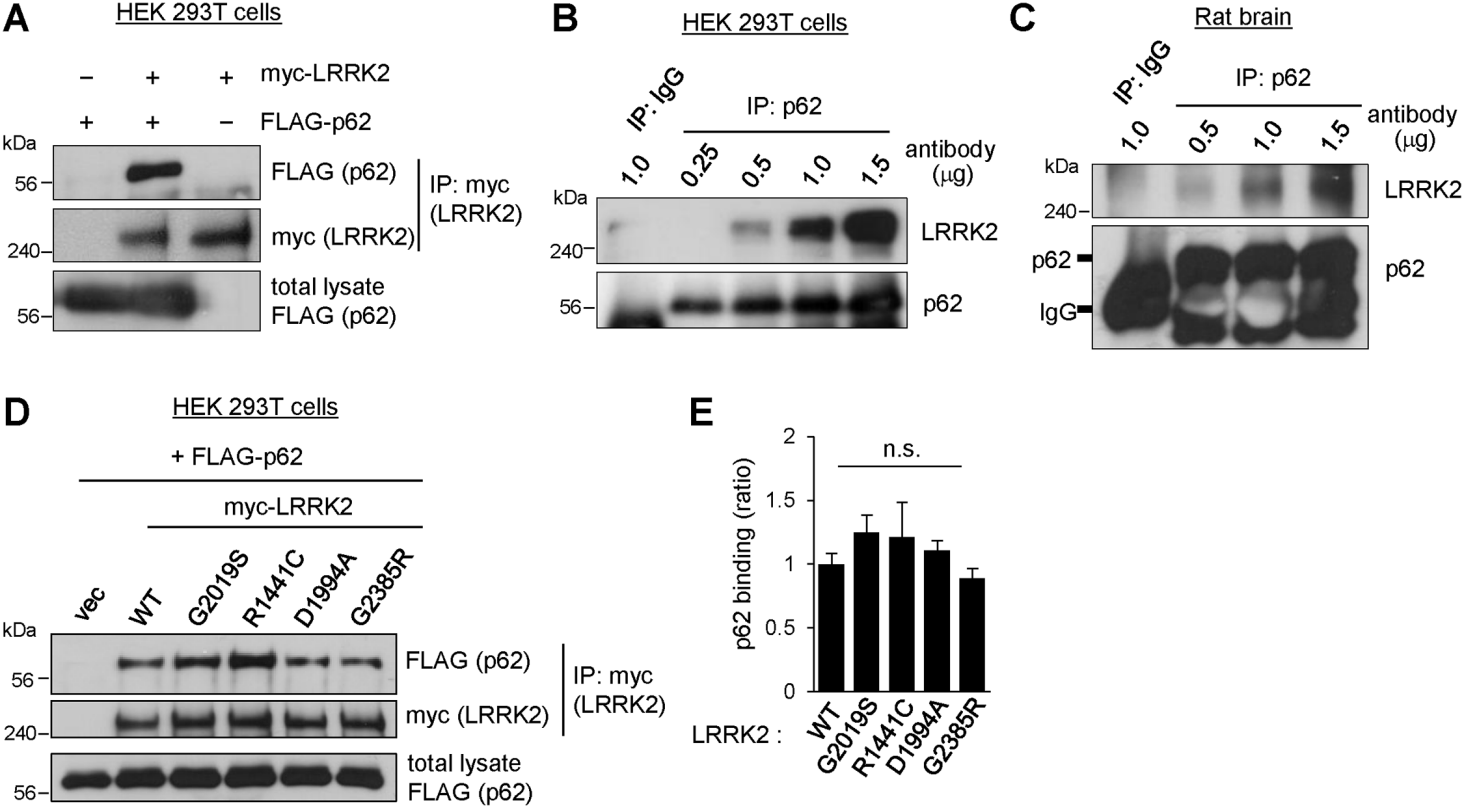
LRRK2 interacts with p62. **A**. HEK 293T cells were transiently co-transfected with FLAG-tagged p62 (FLAG-p62), c-myc-tagged LRRK2 (myc-LRRK2), or both. Twenty-four hours after transfection, total cell lysates were subjected to immunoprecipitation with anti-c-myc monoclonal antibody 9E10. Binding of p62 to LRRK2 was analyzed with western blotting. **B**. Binding of endogenous LRRK2 to p62 was examined in HEK 293T cells via immunoprecipitation with the indicated quantities of anti-p62 antibody (0.25~1.5 μg). **C**. The P2 fraction of total rat brain lysate was subjected to immunoprecipitation with the indicated quantities of anti-p62 antibody (0.5~1.5 μg). Immune complexes were resolved by SDS-PAGE, followed by western blotting against the indicated antibodies. One microgram of rabbit anti-GFP antibody was used as a negative control (IgG). **D**. HEK 293T cells were transiently transfected with myc-tagged LRRK2 WT or mutant expression constructs (G2019S, R1441C, D1994A, or G2385R) and FLAG-tagged p62. Co-immunoprecipitation was performed as shown in panel A. **E**. Bar graph shows the relative binding of p62 to mutant LRRK2, normalized to LRRK2 WT. The data were obtained from four independent experiments; n.s. indicates p > 0.05 versus WT binding, analyzed with one-way ANOVA.

Because p62 is an autophagy adaptor protein that mediates selective turnover of ubiquitinated proteins in autophagy, we postulated that LRRK2 is degraded by a selective autophagy pathway via direct interaction with p62. To explore the direct interaction between p62 and LRRK2, we expressed FLAG-tagged p62 cDNA and c-myc-tagged LRRK2 in HEK 293T cells and performed a co-immunoprecipitation assay. The lysates were immunoprecipitated with anti-myc antibody and probed with anti-FLAG antibody to detect the binding of p62 to LRRK2. We observed robust interaction between LRRK2 and p62, as shown in Fig 2A. Next, we examined the interaction between endogenous p62 and LRRK2 in HEK 293T cells and rat brain. We found that p62 is co-immunoprecipitated with endogenous LRRK2 in an antibody concentration-dependent manner in both HEK 293T cells and rat brain lysates (Fig 2B and 2C).

Unlike wild-type (WT) LRRK2, pathogenic mutant LRRK2 can inhibit the degradation of substrates through the ALP [26]. Therefore, to determine whether the binding of p62 to pathogenic mutant LRRK2 is altered, we used WT LRRK2 and pathogenic LRRK2 variants (G2019S, R1441C, D1994A, and G2385R) found in familial PD patients. G2019S is a kinase domain mutant that exhibits increased kinase activity, R1441C is a ROC domain mutant, D1994A is a COR domain mutant with kinase-dead activity, and G2385R is a WD40 domain mutant. We transfected WT and mutant LRRK2 cDNA along with p62 in HEK 293T cells and analyzed their interactions as shown in Fig 2A. We found that all of the mutant proteins bound to p62; however, we found no significant difference in p62 binding affinity between the mutants and WT LRRK2 (Fig 2D and 2E).

### The N-terminal region of LRRK2 interacts with the SOD mutant interacting region (SMIR) of p62

**Fig 3:**
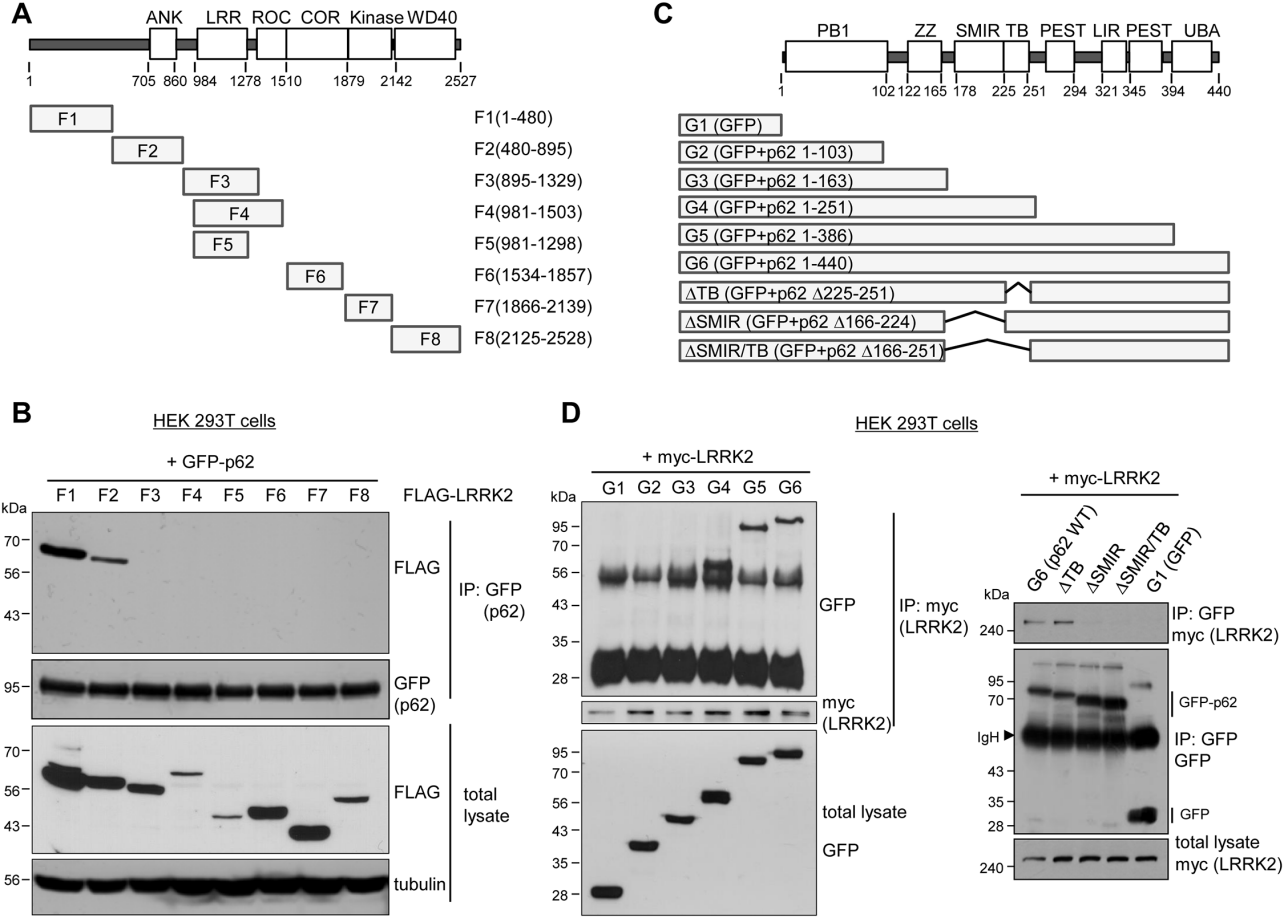
Mapping of the binding domains between LRRK2 and p62. **A**. Schematic representation showing LRRK2 domains and the LRRK2 fragments (F1-F8) used in panel A. F1: amino acids (aa) 1–480, N-terminus segment 1; F2: aa 480–895, N-terminus segment 2; F3: aa 895–1329, N-terminus segment 3; F4: aa 981–1503, LRR + GTPase; F5: aa 981–1298, LRR; F6: aa 1534–1857, COR; F7: aa 1866–2139, kinase; F8: aa 2125–2528, WD40 domain. **B**. p62 interacts the N-terminal region of LRRK2. GFP-tagged p62 was co-transfected with the indicated FLAG-LRRK2 fragment constructs F1–F8 in HEK 293T cells as shown in panel B. Immunoprecipitation was carried out by using rabbit anti-GFP antibody, and the binding domains were detected with mouse anti-FLAG antibody. **C**. Schematic representation showing p62 domains and p62 fragments (G1-G6) used in panel A. G1: GFP alone; G2: aa 1–103, p62 Phox and Bem1 (PB1); G3: aa 1–163, p62 PB1 and zinc finger (ZZ); G4: aa 1–251, p62 ΔLIR/ΔUBA; G5: aa 1–386, p62 ΔUBA; G6: aa 1–440, p62 full length. ΔTB, ΔSMIR, ΔSMIR/TB indicate the deletion mutants of p62 aa 225–251, aa 166–224, aa 166–251, respectively. All mutant constructs were sequence-proved with complete fidelity. LIR, microtubule-associated protein 1A/1B-light chain 3-interaction region; UBA, ubiquitin-associated domain. **D**. LRRK2 interacts with p62 through the SMIR of p62. HEK 293T cells were co-transfected with GFP-p62 fragment constructs (G1-G6) and myc-tagged LRRK2. Co-immunoprecipitation assays were performed using anti-myc (left panel) or anti-GFP antibody (right panel). Arrowhead indicates immunoglobulin heavy chain (IgH).

For determining which LRRK2 domain interacts with p62, FLAG-tagged LRRK2 fragments (generously provided by Dr. Valina Dawson [Johns Hopkins University, MD, USA]) [29] were co-immunoprecipitated with GFP-tagged p62 in HEK 293T cells. LRRK2 fragments designated F1–F8 (Fig 3A) cover the entire domain of the LRRK2 protein. We found that full-length p62 strongly interacts with F1 armadillo (F1) and ankyrin (F2) repeat domains (amino acids 1–895), whereas it fails to interact with the ROC-COR, kinase, or WD40 domains of LRRK2. This result showed that p62 physically interacts with LRRK2 through the N-terminal region of LRRK2 (Fig 3B).

We next sought to identify the p62 domain responsible for the interaction with LRRK2. At least seven functional domains are present in p62 (Fig 3C): N-terminal Phox and Bem1 (PB1); zinc finger (ZZ); SOD mutant interacting region (SMIR); TRAF6 binding (TB); LIR; two PEST sequences rich in proline (P), glutamic acid (E), serine (S), and threonine (T); and C-terminal UBA domain [15, 17, 30]. GFP-tagged fragments of p62 designated G2–G6 (Fig 3C) along with control GFP (G1) were co-immunoprecipitated with LRRK2. We found that full-length (G6), ΔUBA domain (G5), and ΔLIR-UBA (G4) p62 was immunoprecipitated with LRRK2, whereas no interaction was detected between LRRK2 and p62 fragments containing the N-terminal PB1 (G2) or ZZ (G3) domains (Fig 3D). Therefore, amino acids 164–251, which span the SMIR and the TB domain of p62, interact with LRRK2. To confirm which domain interacts with LRRK2, we generated three deletion mutants of GFP-p62, in which both the SMIR and TB domains are deleted (ΔSMIR/TB, amino acids 166–251), the SMIR is deleted (ΔSMIR, amino acids 166–224), or the TB is deleted (ΔTB, amino acids 225–251). We performed co-immunoprecipitation experiment using anti-GFP antibody and probed LRRK2 bound to the deleted mutants. We found that LRRK2 binds to the full length p62 or ΔTB mutant, but not to the ΔSMIR/TB or ΔSMIR mutant, indicating that amino acids 166–224, which span the SMIR of p62, interact with LRRK2 (Fig 3D). Collectively, these results indicate that the N-terminal region of LRRK2 interacts with the SMIR of p62. However, we were not able to exclude the possibility that the interaction between LRRK2 and p62 may be indirect.

### p62 regulates LRRK2 stability via the ALP

**Fig 4:**
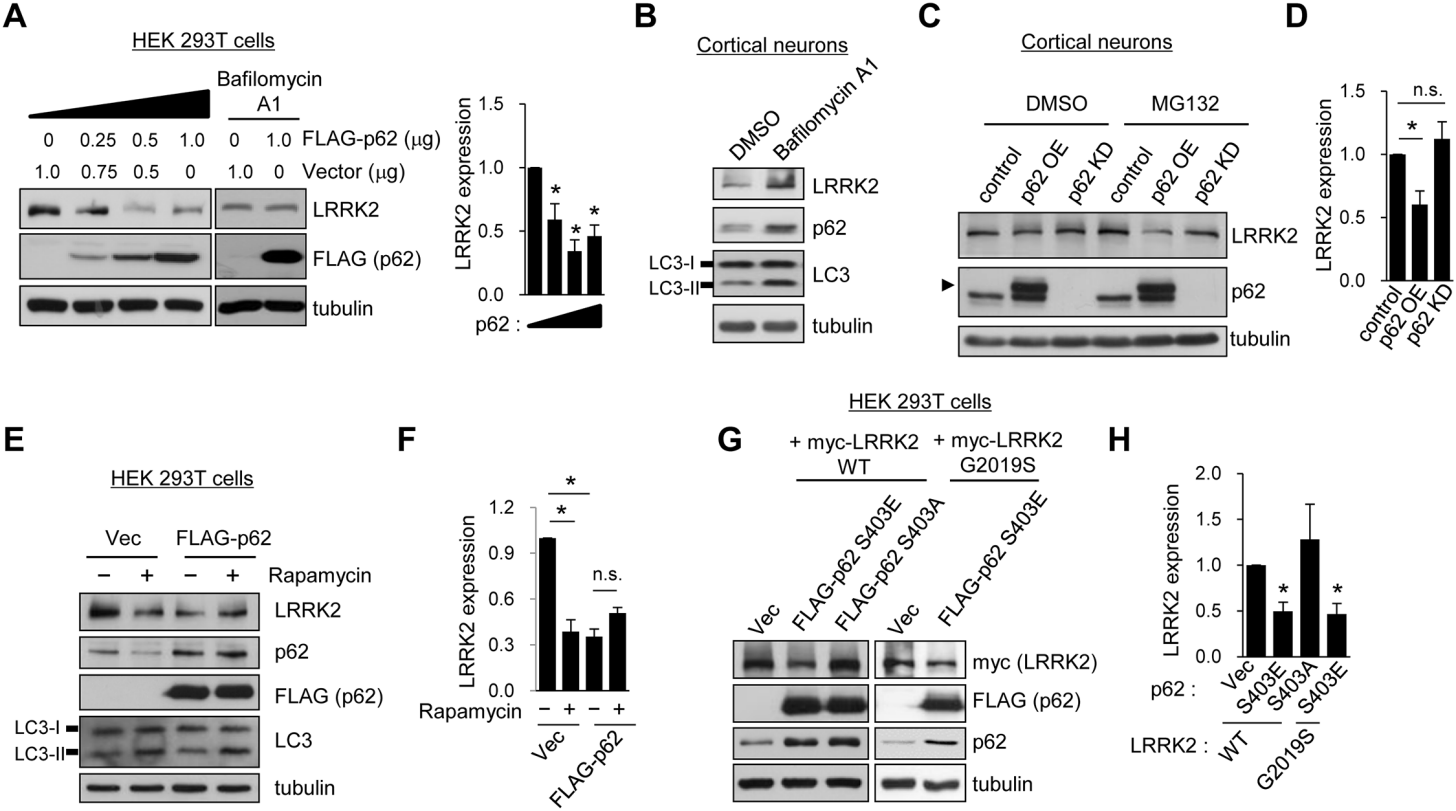
Stability of LRRK2 is regulated by p62. **A**. HEK 293T cells were transfected with 0.25, 0.5, or 1.0 μg FLAG-p62 expression plasmid or control vector for 24 h. Bafilomycin A1 was treated for 2 h. Endogenous LRRK2 expression was detected with western blotting by using LRRK2 antibody from total cell lysates. Quantification data from panel A represent means of LRRK2 expression ± SEM (Vec, 1.00 ± 0.00; 0.25 μg p62, 0.59 ± 0.12; 0.5 μg p62, 0.34 ± 0.15; 1.0 μg p62, 0.46 ± 0.15; n = 3; *p < 0.05, paired *t*-test). **B**. Primary cortical neurons were treated with bafilomycin A1 for 2 h and endogenous protein levels were detected with western blotting using the indicated antibodies. **C**. Primary cortical neurons were infected with lentivirus that overexpress GFP (control) or p62 (p62 OE) under a ubiquitin promoter. Neurons were also infected with lentivirus that harbor p62 small hairpin RNA (shRNA) under an H1 promoter to knock down the expression of p62 (p62 KD). Five to seven days after infection, the neurons were treated with DMSO or 5 μM MG132 for 2 h. Endogenous LRRK2 expression was examined with western blotting. Arrowhead indicates the exogenous expression of 3 X FLAG-tagged p62, in which bands were shifted owing to the increased molecular weight of p62 fused with three tandem FLAG epitopes. **D**. Quantification of band intensity of LRRK2 relative to that of the control is shown. Means ± SEM (control, 1.00 ± 0.00; P62 OE, 0.60 ± 0.11; p62 KD, 1.12 ± 0.49; n = 4 for p62 OE, n = 13 for p62 KD; *p < 0.05, n.s. indicates p > 0.05, paired *t*-test). **E**. A FLAG-p62 expression plasmid was transfected in HEK 293T cells. Twenty-four hours after transfection, the cells were treated with 100 nM rapamycin for 2 h, and protein expression levels were analyzed with western blotting with the indicated antibodies. **F**. Bar graph data represent the means of normalized LRRK2 expression ± SEM (Vec, 1.00 ± 0.00; Vec with rapamycin, 0.39 ± 0.08; p62, 0.35 ± 0.05; p62 with rapamycin, 0.51 ± 0.04; n = 3, *p < 0.01, n.s. indicates p > 0.05, paired *t*-test). **G**. HEK 293T cells were co-transfected with WT or G2019S myc-LRRK2 and phosphomimetic mutant FLAG-p62 Ser403Glu (S403E) or dephosphorylated mutant Ser403Ala (S403A). LRRK2 expression was detected with western blotting. **H**. Bar graph data represent the means of normalized LRRK2 expression ± SEM from panel G (Vec, 1.00 ± 0.00; S403E, 0.50 ± 0.01; S403A, 1.28 ± 0.66; S403E in G2019S, 0.47 ± 0.11; n = 3–4, *p < 0.05, paired *t*-test).

p62 binds to both ubiquitinated proteins and LC3 and is a receptor for the selective transport of polyubiquitinated proteins to autophagosomes. To investigate whether p62 regulates the stability of LRRK2, we overexpressed p62 in HEK 293T cells and examined the endogenous expression levels of LRRK2. We found that dose-dependent expression of p62 for 24 h led to a marked decrease in endogenous LRRK2 protein levels (Fig 4A). The degradation of LRRK2 seemed to reach saturation in autophagosomes when p62 was expressed at levels higher than 0.5 μg in HEK 293T cells. The treatment with bafilomycin A1 inhibited the p62-induced degradation of LRRK2 in HEK 293T cells (Fig 4A). In addition, the treatment with bafilomycin A1 enhanced the expression of LRRK2 and p62 in primary cortical neurons (Fig 4B). These results suggest that p62 plays a role in the degradation of LRRK2 through the macroautophagy pathway (Fig 4A and 4B).

To examine the effect of p62 on LRRK2 stability in neurons, we overexpressed or knocked down p62 in primary cortical neurons by using a lentivirus. We confirmed that lentivirus-mediated overexpression of p62 reduced steady-state levels of endogenous LRRK2 protein in neurons (Fig 4C and 4D). However, knocking down endogenous p62 expression did not change LRRK2 protein levels (Fig 4C and 4D). Proteasomal inhibition with MG132 did not block the degradation of LRRK2 by p62 overexpression, which suggested that the degradation of LRRK2 by p62 is primarily mediated through the ALP (Fig 4C and 4D).

To further explore the role of p62 in LRRK2 degradation, we treated HEK 293T cells with rapamycin, an inducer of autophagy [15, 31]. Rapamycin treatment for 2 h led to a marked reduction of endogenous LRRK2 protein level—approximately 60%—and a substantial decrease in endogenous p62 (Fig 4E and 4F). Overexpression of p62 also caused a sharp reduction in endogenous LRRK2 without additional decrease after rapamycin treatment (Fig 4E and 4F). These results suggest that LRRK2 is degraded by p62 in the rapamycin-induced autophagy.

The phosphorylation of p62 at Ser403 reportedly increases the affinity of polyubiquitnated proteins and enhances their autophagic degradation [32]. To examine the effect of Ser403 phosphorylation on the autophagic degradation of LRRK2, we co-transfected HEK 293T cells with LRRK2 and either p62 Ser403 phosphomimetic mutant Ser403Glu (S403E) or dephosphorylated mutant Ser403Ala (S403A) (Fig 4G and 4H). We found that endogenous LRRK2 was drastically reduced by the S403E mutant p62 but not by the S403A mutant p62, suggesting that phosphorylation of p62 regulates LRRK2 degradation via the ALP. We tested whether mutant LRRK2 and WT LRRK2 are differentially regulated by phosphorylated p62. However, G2019S mutant LRRK2 was also degraded to an extent similar to that of WT LRRK2 by S403E mutant p62 (Fig 4G and 4H).

### LRRK2 indirectly regulates Ser351 phosphorylation of p62

**Fig 5:**
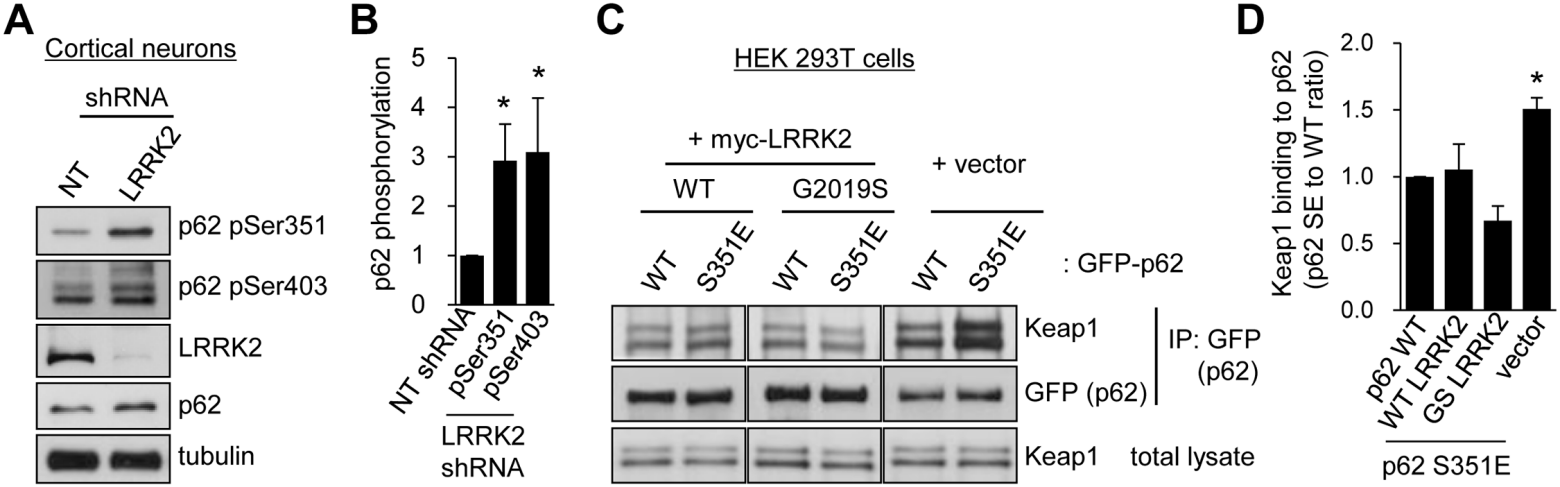
LRRK2 regulates Ser351 phosphorylation of p62. **A**. Primary cortical neurons were infected with lentivirus that contain LRRK2 shRNA or a non-target shRNA sequence (NT shRNA) for 7 days, and endogenous protein expression levels were analyzed via western blotting with the indicated antibodies. **B**. Relative phosphorylation levels of p62 after LRRK2 knockdown normalized to NT shRNA were quantified. Summary data are presented as means ± SEM from three independent experiments (NT shRNA, 1.00 ± 0.00; pSer351, 2.92 ± 0.74; pSer403, 3.10 ± 1.09; n = 3; *p < 0.05, paired *t*-test). **C**. GFP-p62 WT or Ser351Glu (S351E) was co-transfected with vector control, WT, or G2019S myc-LRRK2 in HEK 293T cells. Endogenous Keap1 bindings to p62 were analyzed via immunoprecipitation with anti-GFP (p62) antibody. **D**. Quantification data from panel C represent means of relative Keap1 binding to p62 ± SEM (WT LRRK2, 1.05 ± 0.19; GS LRRK2, 0.67 ± 0.11; vector, 1.51 ± 0.08; n = 3; *p < 0.05, paired *t*-test).

LRRK2 has been implicated in ALP regulation, although the precise links between LRRK2 and macroautophagy have not been identified [33]. Given our findings that p62 binds to LRRK2 and regulates its stability, we hypothesized that LRRK2 regulates the macroautophagy pathway by acting through p62 function. Because phosphorylation of p62 has been proposed to increase the binding affinity for ubiquitin chains [32], we examined whether p62 phosphorylation is altered by LRRK2 knockdown by using lentivirus-mediated shRNA in primary cortical neurons. We observed that both Ser351 and Ser403 phosphorylation of p62 are markedly increased by LRRK2 knockdown in these cells (Fig 5A and 5B).

Previous study has reported that Ser351 phosphorylation of p62 increases the binding affinity of p62 for Keap1 and competitively inhibits Keap1-Nrf2 interaction. Consequently, free Nrf2 can translocate into the nucleus to induce cytoprotective proteins [34]. Consistent with this report, phosphomimetic mutant Ser351Glu (S351E) p62 pulled down Keap1 50% more than WT p62 did in HEK 293T cells (right panel in Fig 5C). Notably, co-expression of the WT or G2019S mutant LRRK2 markedly reduced interaction between phosphorylated p62 and Keap1 to the steady-state level (Fig 5C and 5D). Therefore, the reduced binding of Keap1 to p62 by LRRK2 might render Keap1 more available, facilitate Keap1-Nrf2 interaction, and reduce Nrf2 translocation into the nucleus, thereby impairing the clearance of cytotoxic substances into autophagosomes.

## Discussion

Macroautophagy is a cellular degradation process that involves the formation of autophagosomes, double-layered membrane structures around cargoes such as long-lived proteins or entire organelles [35, 36]. Autophagosomes fuse with lysosomes to degrade their cargoes. Macroautophagy was initially considered a rather nonselective process; however, growing evidence suggests that it is a selective autophagy responsible for the degradation of ubiquitinated proteins or cellular structures through autophagic receptors such as p62 and NBR1 [1, 2]. In the present study, we investigated the role of p62 in the regulation of LRRK2 stability. We demonstrated that p62 has a physical interaction with LRRK2 in which the N-terminal region of LRRK2 binds to the SMIR of p62. We found that LRRK2 is degraded by the overexpression of p62 through the ALP. Furthermore, LRRK2 indirectly regulates Ser351 and Ser403 phosphorylation of p62, and the interaction between the phosphorylated p62 and Keap1 is reduced by LRRK2.

LRRK2 and its mutant are involved in cellular degradation pathways such as the UPS, CMA, and macroautophagy [26, 37, 38]. Moreover, LRRK2 apparently interferes with these degradation pathways by interacting with various pathway proteins. For example, the expression of LRRK2 in A53T α-synuclein transgenic mice accelerates abnormal ubiquitin-positive aggregation containing LRRK2, which may arise when the UPS is impaired [39]. On the contrary, accumulation and aggregation of ubiquitinated proteins have been observed in the kidneys of LRRK2 knockout mice, presumably as consequences of impaired ALP and UPS functioning [40]. We determined that the degradation of WT LRRK2 is blocked by treatment with inhibitors against UPS, CMA, and macroautophagy, which indicates that LRRK2 can be degraded through all of these pathways in neurons (Fig 1).

The results of our study showed that LRRK2 interacts with overexpressed p62 in HEK 293T cells as well as with endogenous p62 in brain (Fig 2). The overexpression of p62 leads to the degradation of LRRK2 by the macroautophagy pathway (see Fig 4A and 4E). However, we were unable to identify significant changes in binding affinity for p62 between WT and mutant LRRK2 (Fig 2D and 2E). Furthermore, phosphorylation of p62, which enhances the autophagic degradation of polyubiquitinated proteins [32], caused no change in the rate of protein degradation of G2019S LRRK2 compared with that of WT LRRK2 (Fig 4G). These findings suggest that p62 is unlikely to mediate cytotoxic effects resulting from insufficient clearance and degradation caused by pathogenic mutations of LRRK2.

Our finding that the overexpression of p62 markedly reduces steady-state levels of LRRK2 protein expression indicates that p62 recruits ubiquitinated LRRK2 for autophagy-lysosomal degradation. However, LRRK2 protein accumulated insignificantly after p62 knockdown, which suggests that the loss of p62 has little effect on the selective autophagy of LRRK2. This lack of phenotype in p62 knockdown might be explained by either the presence of an alternative degradation pathway for LRRK2 or the compensation effect of other autophagy receptors such as NBR1.

Several protein domains in p62 facilitate its cellular functions as a signaling scaffold or a cargo receptor targeting protein aggregates for degradation [8, 41]. By directly binding to LC3 and ubiquitin via its LIR and UBA domains, respectively [13, 34], p62 behaves as a selective autophagy receptor for polyubiquitinated proteins. The activity of p62 in selective autophagy is regulated by its phosphorylation status. Phosphorylation at Ser403 of the p62 UBA domain, which is regulated by casein kinase 2 or TBK1, enhances the binding affinity for ubiquitin and thus promotes the autophagic degradation of polyubiquitinated proteins [32, 42]. Ser351 phosphorylation of the p62 KIR domain contributes to cellular defense mechanisms against oxidative stress by activating the Keap1-Nrf1 pathway [9, 34, 43]. Ser351 phosphorylation increases p62 affinity for Keap1, which competitively releases Nrf2 from Keap1. As a result, Nrf2 translocates into the nucleus and promotes the transcription of multiple Nrf2 target genes encoding antioxidant proteins and anti-inflammatory enzymes, whereas the ubiquitinated cargoes, together with Keap1 and phosphorylated p62, are degraded by autophagy [34]. We found that LRRK2 knockdown increases Ser351 and Ser403 phosphorylation of p62. Furthermore, both WT and G2019S mutant LRRK2 seem to inhibit the Keap1-Nrf1 pathway by increasing the release of Keap1 from phosphorylated p62, thereby enhancing Keap1-Nrf2 interaction and decreasing Nrf2 translocation into the nucleus. Accordingly, it is plausible that LRRK2 interferes with the macroautophagy degradation of cytotoxic protein aggregates and cellular defense mechanisms by regulating the phosphorylation state of p62 and the Keap1-Nrf1 pathway, although we were unable to identify a direct kinase substrate of LRRK2 responsible for p62 phosphorylation. Further study of the detailed mechanisms regulating selective autophagy by p62 in LRRK2 animal models will be invaluable.
